# Supplementary material for: A Secreted Lignin Peroxidase Required for Fungal Growth and Virulence and Related to Plant Immune Response
Source: Int J Mol Sci. 2022 May 28;23(11):6066. doi: 10.3390/ijms23116066 (PMC9181491; doi:10.3390/ijms23116066)
Supplement: Supplementary file 1 [file ijms-23-06066-s001.zip › Supplementary Table S1.pdf]

Supplementary Table S1. Primers used in this study

Primers	Sequence 5´-3´	Purpose
BkLiP1-qF	CAGAAGCACTGGAACGAGGAT	RT-qPCR analysis of *LiPs* in *B. kuwatsukai*
BkLiP1-qR	CACTCGGTGAGGTTGTTGATGT	
BkLiP2-qF	GGGTTGGTTCCTGGATGGTT	
BkLiP2-qR	CGGCATTATCAGAGCGAGTG	
BkLiP3-qF	CCACCATCCTCCTCACTT	
BkLiP3-qR	ATATCGTGGAGGGTGCTT	
BkLiP4-qF	GGCGGTCTGGACGCAAAT	
BkLiP4-qR	GGTGCCTTGGTCGTGGAA	
BkLiP5-qF	CGGTTACCTCACGAGTCTGTT	
BkLiP5-qR	CATAGTGGGCCTGTGCTTCT	
BkLiP6-qF	TTCTTCGCCGACTTCTCCAGC	
BkLiP6-qR	CGCCTCCTCGTTAGCCTTGC	
BkLiP7-qF	ACGATAAGGAAACTGGCACTG	
BkLiP7-qR	GGTAATCCACGGGAACTGC	
18S rRNA-F1	ACGGATCTCTTGGTTCTGG	Internal reference primers of *B. kuwatsukai*
18S rRNA-R1	GCATGCCCTTCGGAATACC	
Sp-*HYG*-F	CTTGGCTGGAGCTAGTGGAGGT	Amplification of *hygromycin* resistance gene
Sp-*HYG*-R	CCCGGTCGGCATCTACTCTATTC	
*HYF*2	CGTTGCAAGACCTGCCTGAA	
*YGR*2	GGATGCCTCCGCTCGAAGTA	
HY-T-R1	GACAGACGTCGCGGTGAGTT	Detection of *hygromycin* resistance gene
HY-T-F1	TCTGGACCGATGGCTGTGTAG	
HYG-177F	AGATCGTTATGTTTATCGGCACT	

HYG-827R	TTGCCGTCAACCAAGCTCT	
BkLiP1-Up1F	AGGTCTCTCCACTATCCCGAAT	Amplification of an upstream fragment of *BkLiP1*
BkLiP1-Up1R	ACCTCCACTAGCTCCAGCCAAGGTGGCGGCTGGTTTGAAA	
BkLiP1-Do1F	GAATAGAGTAGATGCCGACCGGGGGGGACATGCAGTTTTCG	Amplification of a downstream fragment of *BkLiP1*
BkLiP1-Do1R	CTCTTAATGAAGTCCAGTGG	
BkLiP1-Up2F	TAACCTGCTCCTGATGAGGCA	Amplification of an internal fragment of *BkLiP1*
BkLiP1-Do2R	CCCTTCTCGGACGTCTTCCT	
det-*BkLiP1*F	ACGGTGATTCCAACCCTGAG	Detection of *BkLiP1* mutants
det-*BkLiP1*-SPF	CATCACGAGCGTTGCTCTCAC	
det-*BkLiP1*R	GAGGCGGGTGTAGGAAGTAG	
da-*BkLiP1*F	ATGGCATGAGGGGTATCCAA	
da-*BkLiP1*R	ACACAAAACGCCAGCCCTAT	
BkLiP2-Up1F	ATCGTCTCCTCATCGGAAAG	Amplification of an upstream fragment of *BkLiP2*
BkLiP2-Up1R	ACCTCCACTAGCTCCAGCCAAGTGACAGTATGTAGAGACG	
BkLiP2-Do1F	GAATAGAGTAGATGCCGACCGGGAGATGACGCCCGCTGATT	Amplification of a downstream fragment of *BkLiP2*
BkLiP2-Do1R	GAAAGCGCATATCACCTCAT	
BkLiP2-Up2F	ACTCTTGCCTGCTCCAATCA	Amplification of an internal fragment of *BkLiP2*
BkLiP2-Do2R	ACAACAGCTGCTCATCCTGGCT	
det-*BkLiP2*F	ATGCACTTCTCCAAGTCCTCC	Detection of *BkLiP2* mutants
det-*BkLiP2*R	AATCCTGTCCGCAATTGGTG	
da-*BkLiP2*F	ACCGCCCCTCTCTATCGTTT	
da-*BkLiP2*R	CATAGTTTCGGCAGGTATCAAT	
C-*BkLiP1*-mspF	GTTTTCAAACCAGCCGCCACCATGTACCCGGGCATGGCCAAC	Amplification of a complementary fragment of *BkLiP1* and *BkLiP1*ΔSP
C-*BkLiP1*-mspR	GGTGGCGGCTGGTTTGAAAAC	
C-*BkLiP1*-Up2F	ATGTCGACCAACTCTTCTCC	

C-*BkLiP1*-Up3F	AAGAAGGCCGAGTACAACCG	
C-*BkLiP1*-Up1R	GTGCTCCTTCAATATCATCTTCTGCTTTACTCGTCCTTGAACA	
C-det-UR	CAGTCATAGCCGAATAGCCT	Detection of *neomycin* resistance gene
C-det-DF	ACTGTTCGCCAGGCTCAAG	
Neo-F	TGCGTTTGTCAAGCAAGGTA	
Neo-R	AGCCAACGCTATGTCCTGAT	
C-*Neo*-F	CAGAAGATGATATTGAAGGAGCAC	Amplification of *neomycin* resistance gene
C-*Neo*-R	ATCCTCAGAAGAACTCGTCA	
Neo-F	TGCGTTTGTCAAGCAAGGTA	
C-*eoR*	GATCGACAAGACCGGCTTCCAT	
Pear-*Actin*-F	CTCCCAGGGCTGTGTTTCCTA	Internal reference primers of *P. pyrifolia*
Pear-*Actin*-R	CTCCATGTCATCCCAGTTGCT	
ClaI-*BkLiP1*F	CCATCGATATGAAGTTCTCCGCAGTCATC	Construction of PVX vector
ClaI-*BkLiP1*-mspF	CCATCGATATGTATCCGGGCATGGCCAACGTTA	
SmaI-*BkLiP*1R	TCCCCCGGGCTCGTCCTTGAACAAGTTCCA	
PVX-F	CAATCACAGTGTTGGCTTGC	
PVX-R	GACCCTATGGGCTGTGTTG	
KpnI-eGFP-F	GGGGTACCATGGTGAGCAAGGGCGAGGA	Construction of pCETNS4 vector
SmaI-eGFP-R	TCCCCCGGGCTTGTACAGCTCGT	
SpeI-*BkLiP1*-F	GGACTAGTATGAAGTTCTCCGCAGTCAT	
SpeI-*BkLiP1*-mspF	GGACTAGTATGTATCCGGGCATGGCCAACGT	
KpnI-*BkLiP1*-R	GGGGTACCCTCGTCCTTGAACAAGTTCC	
KpnI-mCherry-F	GGGGTACCATGGTGAGCAAGGGCGAGGA	
SmaI-mCherryNLS-R	TCCCCCGGGCACCTTACGCTTCTTCTTCGGCTTGTACAGCTC	
EcoRI-*BkLiP1*-SP-F	CGGAATTCATGAAGTTCTCCGCAGTC	Construction of pSUC2 vector

XhoI-*BkLiP1*-SP-R	CCCTCGAGAGCAAGGGCCGGCTGGAG	
XbaI-*BkLiP1*F	GCTCTAGAATGAAGTTCTCCGCAGTCATC	
XbaI-*BkLiP1*-mspF	GCTCTAGAATGTATCCGGGCATGGCCAACGTTA	Construction of pCNF3-YFP vector
BamHI-*BkLiP1*R	CGGGATCCCTCGTCCTTGAACAAGTTCCA	
NbActin-qF	TGGTCGTACCACCGGTATTGTGTT	
NbActin-qR	TCACTTGCCCATCAGGAAGCTCAT	
NbHIN1-qF	CCAACTTGAACGGAGCCTATTA	
NbHIN1-qR	AGGCATCCAAAGAGACAACTAC	
NbHSR203J-qF	ACGCAGATTTCAACCGAGTAT	
NbHSR203J-qR	GCCAGTCGCATTGGAGATAA	
NbPR1α-qF	CCGCCTTCCCTCAACTCAAC	
NbPR1α-qR	GCACAACCAAGACGTACTGAG	
NbPR2-qF	AGGTGTTTGCTATGGAATGC	
NbPR2-qR	TCTGTACCCACCATCTTGC	RT-qPCR analysis of Marker genes in *N. benthamiana*
NbPR4-qF	GGCCAAGATTCCTGTGGTAGAT	(Zhang et al 2021)
NbPR4-qR	CACTGTTGTTTGAGTTCCTGTTCCT	
NbLOX-qF	AAAACCTATGCCTCAAGAAC	
NbLOX-qR	ACTGCTGCATAGGCTTTGG	
NbERF1-qF	GCTCTTAACGTCGGATGGTC	
NbERF1-qR	AGCCAAACCCTAGCTCCATT	
CYP71D20-qF	AAGGTCCACCGCACCATGTCCTTAGAG	
CYP71D20-qR	AAGAATTCCTTGCCCCTTGAGTACTTGC	
NbPti5-qF	CCTCCAAGTTTGAGCTCGGATAGT	
NbPti5-qR	CCAAGAAATTCTCCATGCACTCTGTC	

NbAcre31-qF	AATTCGGCCATCGTGATCTTGGTC
NbAcre31-qR	GAGAAACTGGGATTGCCTGAAGGA
NbWRKY7-qF	CACAAGGGTACAAACAACACAG
NbWRKY7-qR	GGTTGCATTTGGTTCATGTAAG
NbWRKY8-qF	AACAATGGTGCCAATAATGC
NbWRKY8-qR	TGCATATCCTGAGAAACCATT

Note: Underline for restriction site and *italics* for genes or species.
