# Supplementary figures and images for: A Secreted Lignin Peroxidase Required for Fungal Growth and Virulence and Related to Plant Immune Response
Source: Int J Mol Sci. 2022 May 28;23(11):6066. doi: 10.3390/ijms23116066 (PMC9181491; doi:10.3390/ijms23116066)

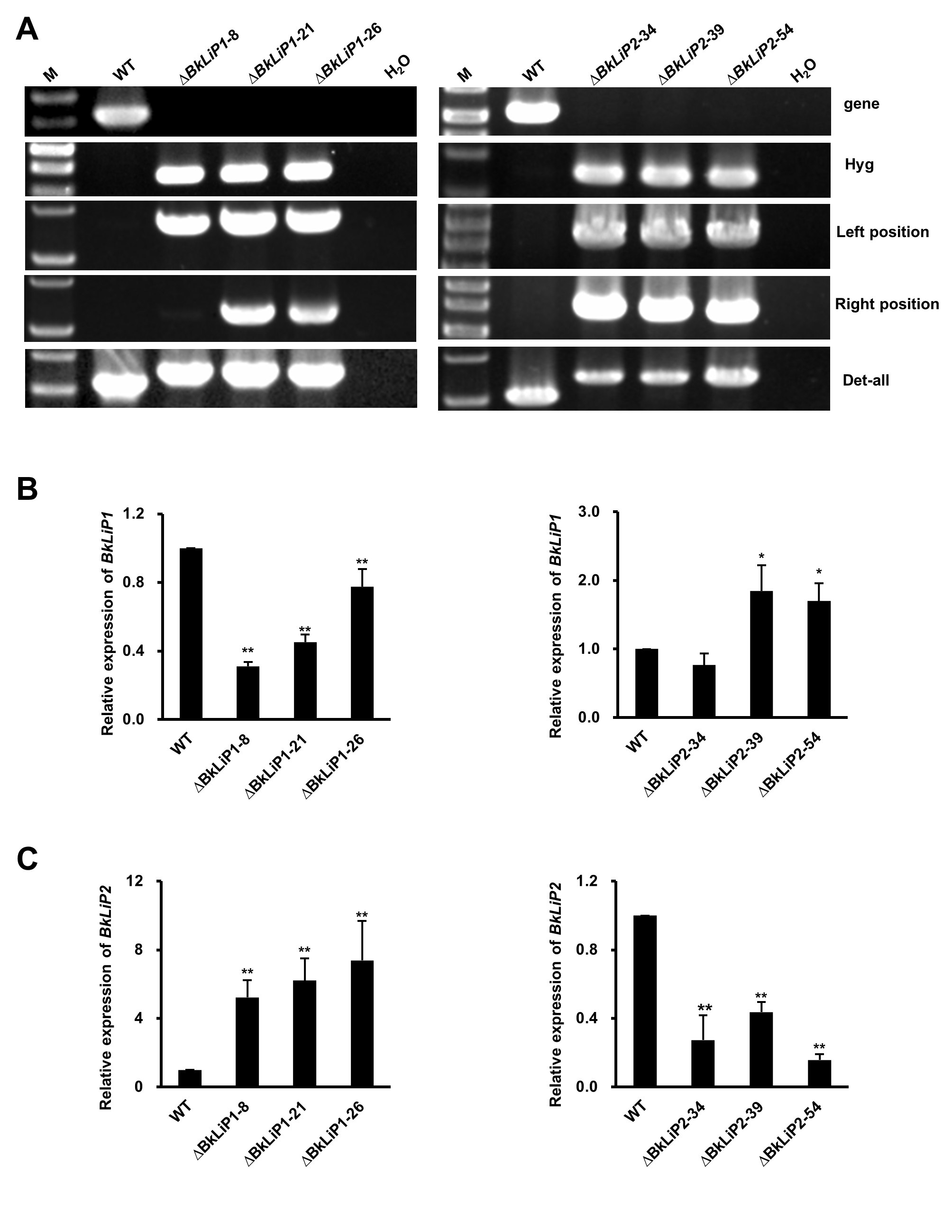

Supplement: Supplementary file 1 [file ijms-23-06066-s001.zip › Supplementary Figure S1.tif]

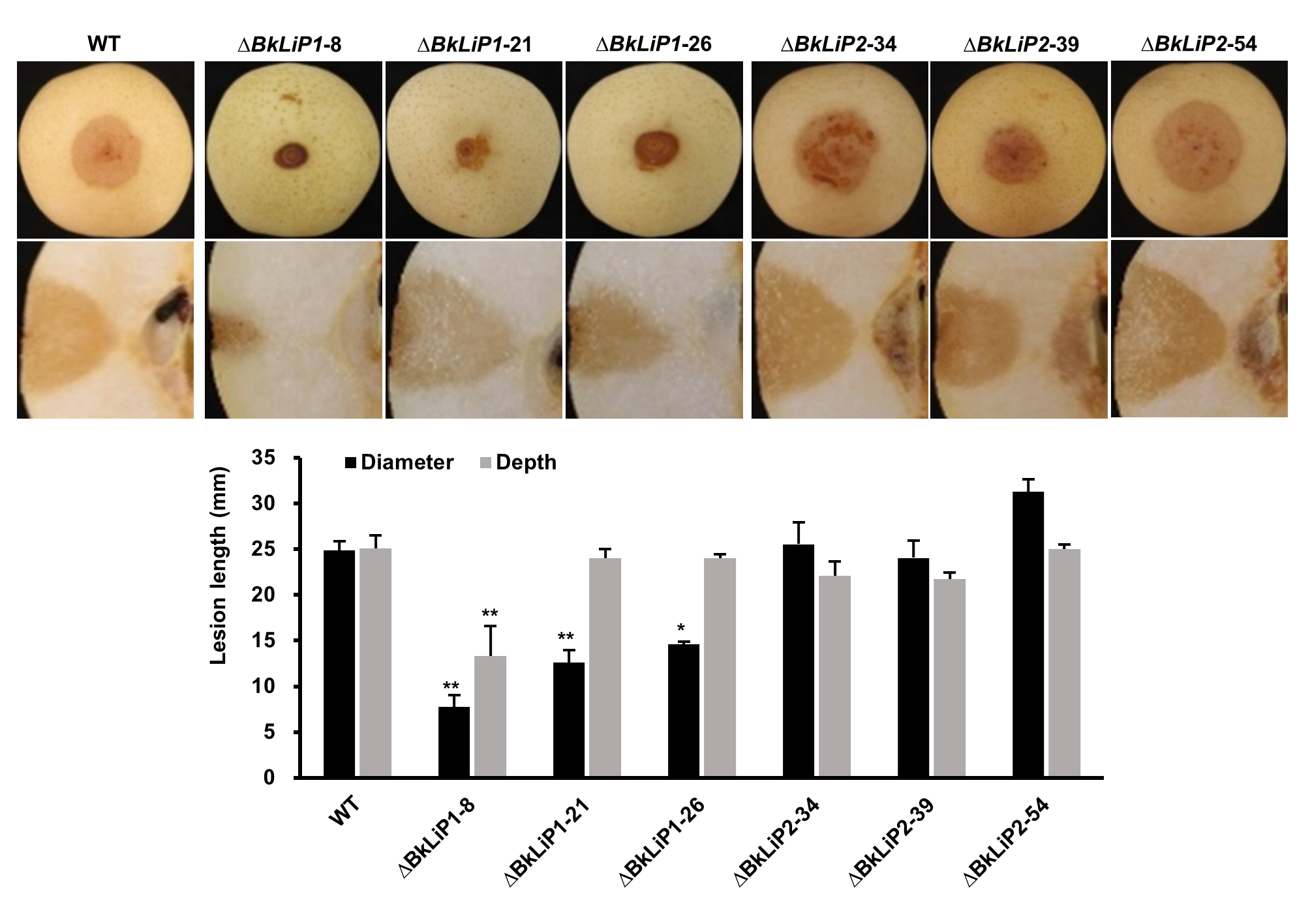

Supplement: Supplementary file 1 [file ijms-23-06066-s001.zip › Supplementary Figure S2.tif]

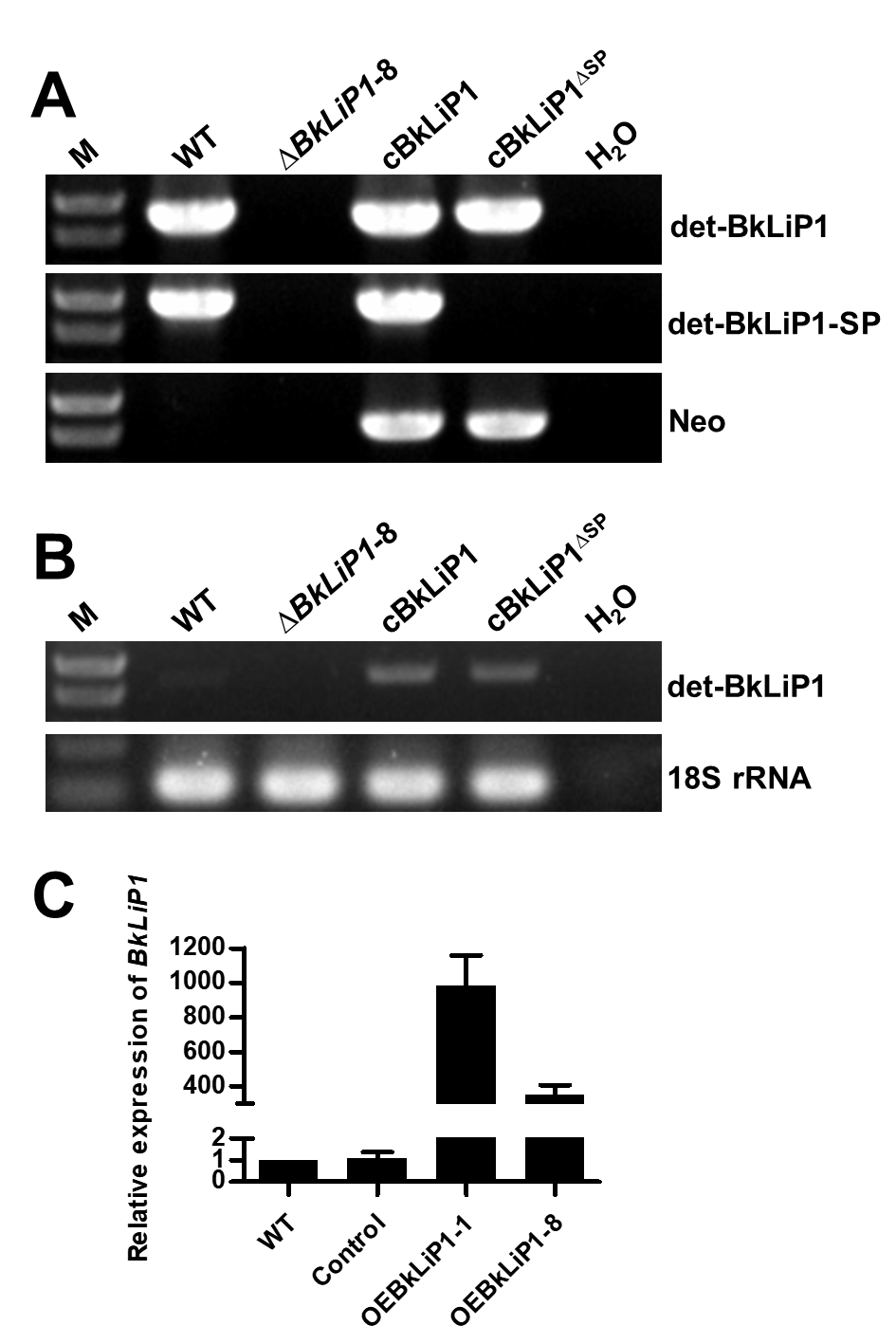

Supplement: Supplementary file 1 [file ijms-23-06066-s001.zip › Supplementary Figure S3.tif]

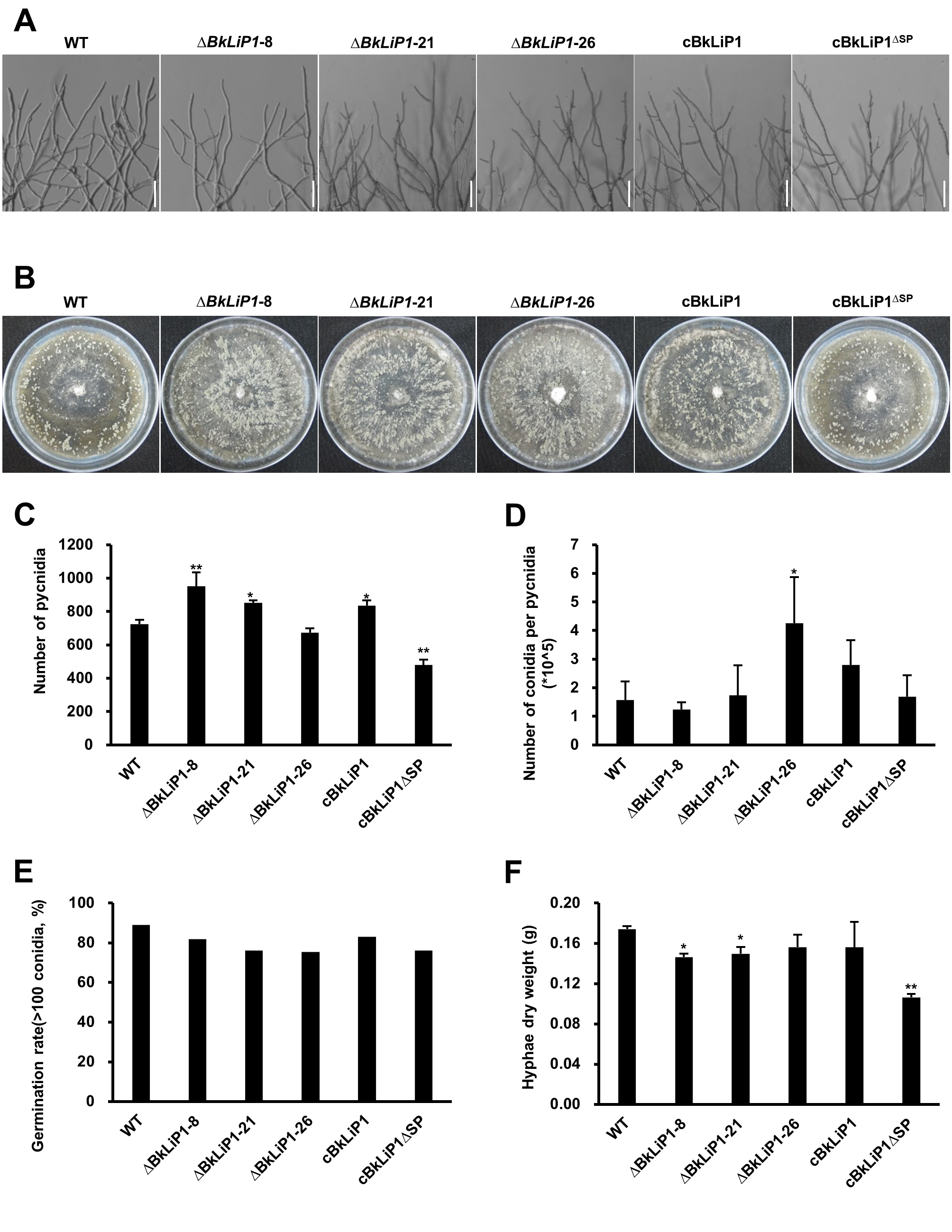

Supplement: Supplementary file 1 [file ijms-23-06066-s001.zip › Supplementary Figure S4.tif]

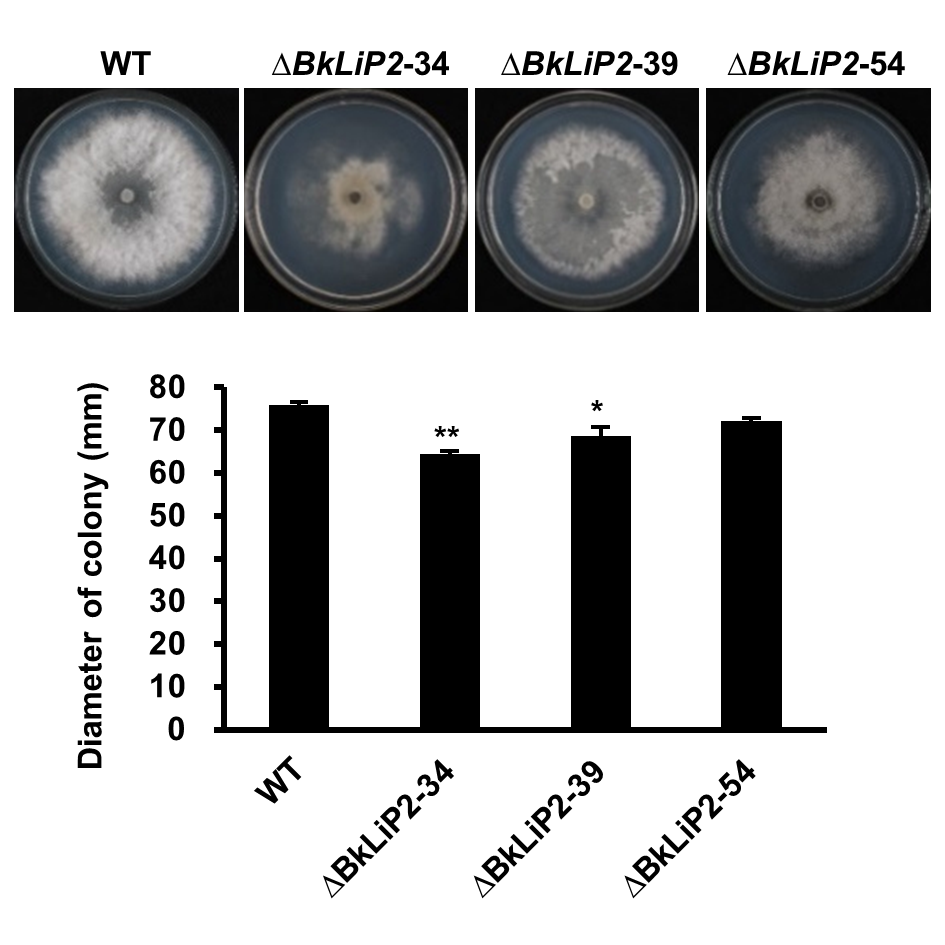

Supplement: Supplementary file 1 [file ijms-23-06066-s001.zip › Supplementary Figure S5.tif]

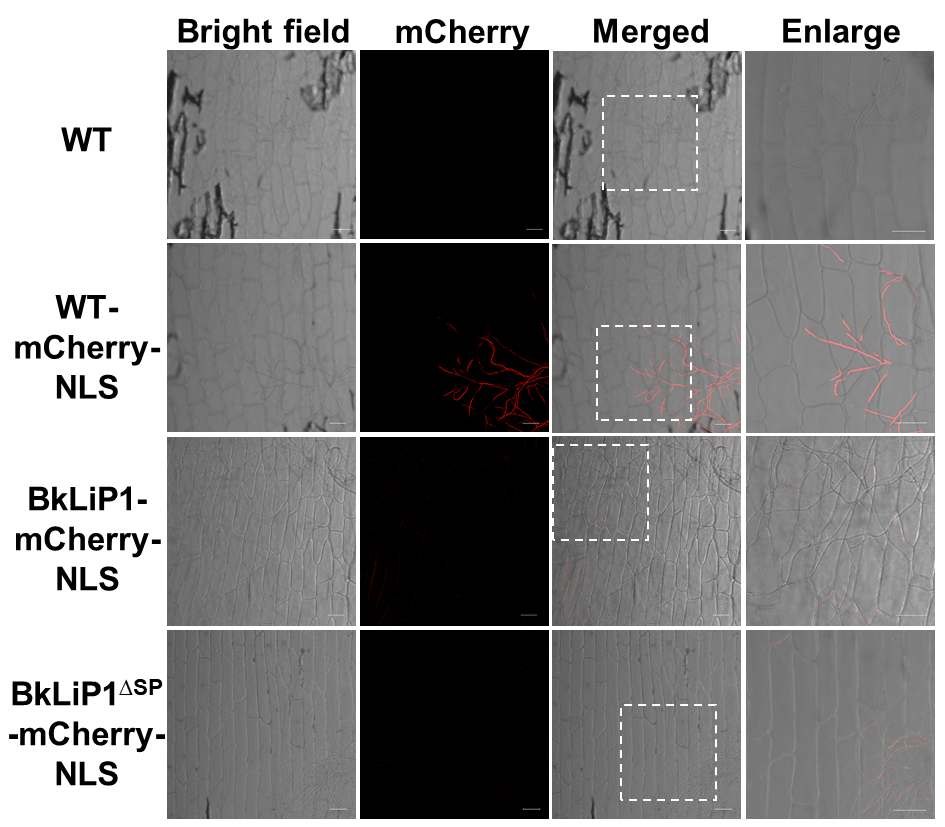

Supplement: Supplementary file 1 [file ijms-23-06066-s001.zip › Supplementary Figure S6.tif]

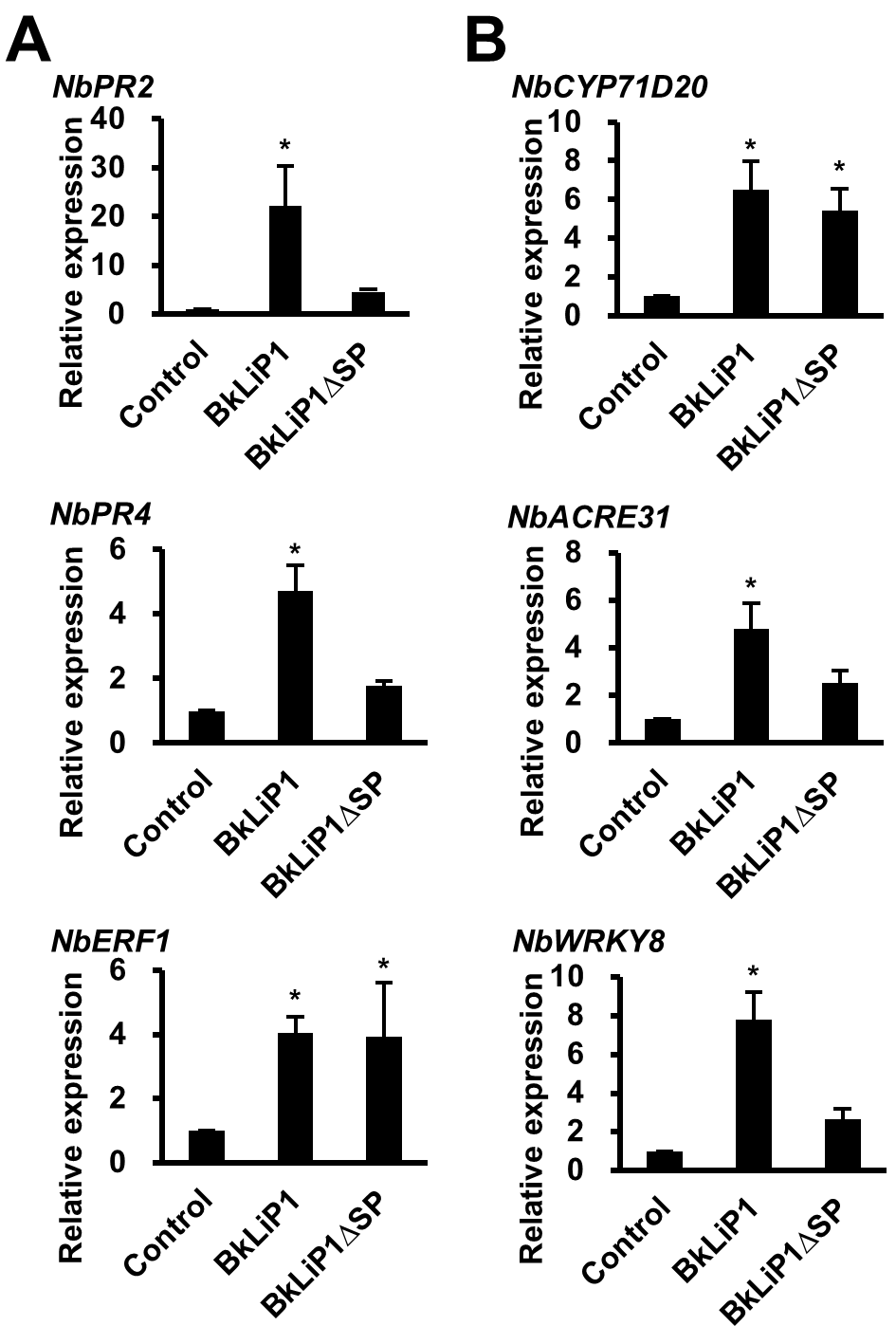

Supplement: Supplementary file 1 [file ijms-23-06066-s001.zip › Supplementary Figure S7.tif]
